# Breaking bad news: A mix methods study reporting the need for improving communication skills among doctors in Pakistan

**DOI:** 10.1186/s12913-024-11056-2

**Published:** 2024-05-06

**Authors:** Muhammad Ahmed Abdullah, Babar Tasneem Shaikh, Kashif Rehman Khan, Muhammad Asif Yasin

**Affiliations:** 1 Health Services Academy, Park Road, Chak Shahzad, Islamabad, 44000 Pakistan; 2 Federal Polyclinic Hospital, Islamabad, Pakistan; 3 Indus Healthcare Network, Islamabad, Pakistan

**Keywords:** Breaking Bad News, Communication skills, Counselling, Physicians’ training, Pakistan

## Abstract

**Background:**

Effective skills and training for physicians are essential for communicating difficult or distressing information, also known as breaking bad news (BBN). This study aimed to assess both the capacity and the practices of clinicians in Pakistan regarding BBN.

**Methods:**

A cross-sectional study was conducted involving 151 clinicians. Quantitative component used a structured questionnaire, while qualitative data were obtained through in-depth interviews with 13 medical educationists. The responses were analyzed using descriptive statistics and thematic analysis.

**Results:**

While most clinicians acknowledged their responsibility of delivering difficult news, only a small percentage had received formal training in BBN. Areas for improvement include time and interruption management, rapport building, and understanding the patients’ point of view. Prognosis and treatment options were not consistently discussed. Limited importance is given to BBN in medical education.

**Discussion:**

Training in BBN will lead to improved patient and attendants’ satisfaction, and empathetic support during difficult times.

**Supplementary Information:**

The online version contains supplementary material available at 10.1186/s12913-024-11056-2.

## Introduction

The duties of physicians extend beyond providing an effective treatment to patients; they also encompass the development of strong communication skills and the establishment of trust with their patients [1]. This emphasis on communication is crucial as it enables patients to cope with the seriousness and severity of their illnesses, to make informed decisions regarding treatment options, and to manage potential side effects [2]. In recent years, there has been a shift in medical practice from a doctor-centered approach to a patient-centered one, where patients play a significant role in the decision-making process, ultimately leading to increased patient satisfaction [3]. However, physicians may find themselves burdened when faced with the task of breaking bad news, fearing the potential reactions of their patients [4, 5]. Neglecting to address this challenge can have negative consequences in terms of patient-centered healthcare, as physicians’ reluctance to disclose a bad news may compromise mental and physical well-being of the patients, and at times of the family members too [6]. On the other hand, physicians are being uncomfortable with their own emotions and do not have enough coping skills to manage their emotions in the moment [7].

Research studies have documented the lack of training and protocols among doctors for breaking bad news. For instance, a research from Brazil revealed that none of the clinicians at a university hospital were aware of any specific protocol or guidelines for this purpose [5]. Similarly, in Canada and South Korea, physician training in breaking bad news is reported to be insufficient, and in many underdeveloped countries, it is virtually non-existent despite curricular reforms [8]. In Northern Portugal, a significant number of family physicians expressed apprehension about breaking bad news and deemed training in this area necessary [9]. In Iran, inadequate training was identified as the main reason behind physicians’ difficulty and fear in delivering bad news to patients, emphasizing the need for formal training in this domain [1]. In India, one research documented diverse opinions among oncologists regarding breaking bad news and sharing information with patients, accenting the necessity for physician training in this aspect [10]. Additionally, a study conducted in Pakistan identified a common reason for increasing violence against healthcare providers as the failure to communicate bad news in a timely and appropriate manner, highlighting the need for better preparation and communication skills during this process [4]. Several protocols and guidelines have been developed for breaking bad news, with the SPIKES protocol being one of the most widely used due to its comprehensive coverage of essential aspects, particularly the emotional aspect of the process [11]. This Six-Step Protocol for Delivering Bad News is SPIKES: S for setting up the meeting, P is assessing the patient’s perception, I for achieving the patient’s invitation, K is providing knowledge and information to the patient, E is addressing the patient’s emotions with empathic responses and S for strategy and summary.

Despite the recommendations of the Pakistan Medical and Dental Council to incorporate communication skills into formal medical curricula, and the ongoing discussions regarding medical curricular reforms in Pakistan over the past two decades, little progress has been made in this regard. This lack of action is evident from a recent study conducted in Peshawar, Pakistan [12]. Thus, the aim of our study was to assess the training as well as the practices of clinicians in Pakistan regarding BBN and provide recommendations for improvement.

## Methods

### Study design

This mixed methods study utilized a cross-sectional design to assess the training and practices of doctors in BBN. The study was conducted at five tertiary care hospitals located in the twin cities of Islamabad and Rawalpindi, namely, Akbar Niazi Teaching Hospital, Benazir Bhutto Hospital, Holy Family Hospital, NESCOM Hospital, and Combined Military Hospital. The data collection period was eight weeks in the first quarter of 2023 to ensure an adequate sample size and data representation. The study participants selected through a simple random sampling included medical personnel directly involved in healthcare delivery within the selected hospitals with a minimum of six months of clinical experience. Medical students and Basic Health Sciences faculty were excluded from the study sample.

### Data collection

To collect the necessary data, a 25-item self-administered questionnaire was developed. The questionnaire encompassed two main sections. The first section focused on recording participants’ demographic information, including age, gender, designation, specialty, and years of experience. This section aimed to establish a comprehensive profile of the participating doctors, providing a contextual background for the subsequent analysis of their responses. The second section of the questionnaire delved into the participants’ knowledge and practices related to breaking bad news, drawing from the established SPIKES protocol [11]. This section comprised a series of questions designed to assess the doctors’ familiarity with the protocol, their adherence to its guidelines, and their overall comfort level in delivering challenging news to patients and their families. The SPIKES protocol, which stands for Setting, Perception, Invitation, Knowledge, Emotions, and Strategy, is a widely recognized framework for effective communication during difficult conversations. Before administering the questionnaire, a pilot study was conducted with ten doctors working in general practice clinics, in Rawalpindi/Islamabad, to ensure its clarity, comprehensibility, and relevance to the research objectives. Feedback from the pilot study participants was incorporated into the final version of the questionnaire to enhance its validity and reliability.

### Sample size calculation

The sample size for this study was determined based on a 95% confidence level, considering a hypothesized population proportion of 11% with a 5% margin of error. The anticipated frequency of this outcome factor was derived from a previous study [13]. The population size was estimated to be 200,000. Using the formula for sample size calculation for frequency in a population (n = [DEFF * N * p * (1-p)] / [(d^2 / Z^2) * (N-1) + p * (1-p)]), where DEFF represents the design effect, N is the population size, p is the hypothesized proportion, d is the margin of error, and Z is the critical value corresponding to the desired confidence level, the required sample size was determined to be approximately 151 participants.

### Data analysis and synthesis

After data collection, the collected data were subjected to comprehensive analysis using SPSS version 22.0. Descriptive statistics, such as frequencies and percentages were computed to summarize the data and gain insights into the training and practices of doctors in breaking bad news.

The qualitative part of the study aimed to gain insights into the practices and challenges associated with breaking bad news in a healthcare setting. The qualitative data were gathered through in-depth interviews with 13 medical educationists from Pakistan. Each interview lasted between 30 and 45 min and took place in the office spaces of the participants to ensure privacy and confidentiality. The participants were individuals who had been involved in teaching medicine for at least 5 years, including 6 clinicians, 4 individuals from medical education, and 3 from basic sciences departments. The interviews were conducted by the principal investigator along with a medical student who accompanied as a note-taker. Rigorous note-taking was done during the interviews to capture detailed information, and where possible, the interviews were audio recorded and later transcribed for analysis. The Braun and Clarke’s thematic analysis method was used as an iterative process which consisted of six steps: (1) becoming familiar with the data, (2) generating codes, (3) generating themes, (4) reviewing themes, (5) defining and naming themes, and (6) locating exemplars [14]. The analysis was conducted by carefully reading and familiarizing with the interview transcripts. Codes were generated to label and categorize meaningful segments of data, which were refined and grouped into broader themes. The research team engaged in discussions to validate the emerging themes and ensure the reliability of the analysis.

## Results

Demographic data of the participants showed that out of the total 151 respondents males were greater in number than females (62.3%), mean age was 30.7(± 8.6 SD) years and the proportion of house officers was the highest, as shown in Table 1. Response rate of the employees of private hospitals was higher than that of the public sector and there were graduates from several medical institutions all over Pakistan.

**Table 1:** Basic demographics of participants

Demographics	Frequency and percentage
**Gender**	
Male	94 (62)
Female	57 (38)
**Specialty**	
Surgery and Allied	60 (40)
Medicine and Allied	54 (36)
Obstetrics and Gynecology	14 (9)
Dentistry	19 (13)
Pediatrics	4 (3)
**Designation**	
House Officer	74 (49)
Medical Officer	24 (16)
PG trainee	30 (20)
Registrar	3 (2)
Consultant	20 (13)
**Organization**	
Public	72 (48)
Private	79 (52)

Table 2 illustrates the responses to various questions related to BBN. Out of the total respondents, 74% reported that BBN was included in their daily duties, indicating that a significant majority of doctors in Pakistan are involved in delivering difficult news to their patients. However, only 9% of the participants reported receiving training specifically focused on BBN, while the remaining 91% had not received such training.

When considering the tenure of the BBN training, a small percentage of doctors (2%) reported receiving training during their MBBS education, followed by 3% during their house job, and 3% during postgraduate training. Surprisingly, the majority of respondents (92%) relied on personal experience rather than formal training to navigate the challenges of BBN. Regarding the availability of formal guidelines for BBN, only 10% of the participants reported having access to such guidelines, while the majority (90%) did not have formal guidelines to follow.

Maintaining privacy during the process of BBN was reported by 14% of the participants, indicating that privacy considerations may not be adequately addressed in some healthcare settings. Similarly, patient attendants’ involvement during the BBN was reported by 78% of the respondents, suggesting that involving family members or caregivers in the process is common.

When it comes to communication techniques during BBN, 64% of doctors reported sitting while delivering the news, while 36% did not. Time and interruption management, rapport building, patient perception exploration, and adequate patient speaking time were areas where improvements were needed, as reported by the participants.

Furthermore, while 52% of the respondents reported avoiding excessive bluntness and handling emotions appropriately, a considerable portion (48%) did not prioritize these aspects. Identification of emotional state, empathic response, and providing time for personal expression were areas where improvements were necessary, as reported by the participants. Moreover, the participants acknowledged the importance of avoiding jargon and technical terms (44%) and breaking the information into small chunks (45%) to enhance patient understanding. However, further efforts were needed to ensure that hopelessness was avoided during the conversation (50%).

Regarding prognosis and treatment options, 20% of the doctors reported discussing these aspects during BBN conversations, indicating that there is room for improvement in ensuring comprehensive information delivery and empathetic counseling.

**Table 2:** Frequencies of responses related to training on breaking bad news among doctors in Pakistan

	Questions	*n* = 151	
		Yes	No
1	Is breaking bad news included in your daily duties?	111 (74%)	40 (26%)
2	Have you ever received a formal training on breaking bad news to patients and their families?	14 (9%)	137 (91%)
3	If yes, what was the tenure of BBN training?	MBBS 03 (2%)	
		House job 04 (3%)	
		Postgraduate training 05 (3%)	
		Personal experience 139 (92%)	
4	Do you follow the formal guidelines for BBN?	15 (10%)	136 (90%)
5	Do you ensure privacy while BBN?	21 (14%)	130 (86%)
6	Do you involve patient’s attendants during BBN?	118 (78%)	33 (22%)
7	Do you make sure that the person in front is sitting while BBN?	97 (64%)	54 (36%)
8	Do you take care of time and interruption during BBN?	39 (26%)	112 (74%)
9	Do you do rapport building before BBN?	32 (21%)	119 (78%)
10	Do you explore patient’s perception about his/her condition before BBN?	41 (27%)	110 (73%)
11	Do you give adequate speaking time to patient during BBN?	26 (17%)	125 (83%)
12	Do you seek patient’s permission before BBN?	50 (33%)	101 (67%)
13	Do you initiate BBN with a warning phrase?	63 (42%)	88 (58%)
14	Do you take care of the level of comprehension of the person in front?	73 (48%)	78 (52%)
15	Do you avoid excessive bluntness during BBN?	78 (52%)	73 (48%)
16	Do you try to avoid jargons and technical terms during BBN?	67 (44%)	84 (56%)
17	Do you provide small chunks of information?	68 (45%)	83 (55%)
18	Do you attempt to avoid hopelessness while BBN?	75 (50%)	76 (50%)
19	Do you try to handle emotions during BBN?	78 (52%)	73 (48%)
20	Are you able to identify the emotional state of the person in front?	50 (33%)	101 (67%)
21	Do you ensure giving time to patient or attendant for personal expression?	21 (14%)	130 (86%)
22	Are your responses empathic during BBN?	68 (45%)	83 (55%)
23	Do you give a prognosis and treatment options?	30 (20%)	121 (80%)
24	Do you summarize and give a strategy to move forward after BBN?	78 (52%)	73 (48%)

In summary, the results highlight several areas where training and guidelines for BBN in Pakistan can be improved. The majority of doctors rely on personal experience rather than formal training, indicating a need for structured educational programs and guidelines in this critical area of healthcare communication. Privacy considerations, effective communication techniques, and emotional support for patients were identified as areas that require further attention and development. The findings emphasize the importance of enhancing training and providing formal guidelines to equip doctors with the necessary skills and strategies for delivering difficult news effectively and compassionately.

The qualitative component of the study involved in-depth interviews with 13 medical educationists from Pakistan. These interviews aimed to explore the level and standard of training on BBN in the curriculum and training of doctors in Pakistan. The interviews revealed several key themes that shed light on the current state of training and education in this area.

### Theme 1: ambiguity in subject domains and integration of communication skills

The medical educationists expressed concerns regarding the lack of clarity in subject domains and the integration of communication skills into the medical curriculum. They suggested that communication skills, including BBN, should be incorporated into the community medicine curriculum. Furthermore, they proposed the introduction of family medicine as a dedicated subject at the undergraduate level, which would provide comprehensive training in communication skills and prepare doctors to handle sensitive conversations effectively.

One interviewee highlighted, *“There is a lack of clarity when it comes to subject domains and the inclusion of communication skills in our medical curriculum. We believe that communication skills, including breaking bad news, should be integrated into the community medicine curriculum. Additionally, introducing family medicine as a dedicated subject at the undergraduate level would ensure that doctors receive extensive training in effective communication, addressing the emotional needs of patients and their families.”* [P6].

This theme emphasizes the need for clear subject domains and the integration of communication skills including BBN within medical education. The proposal to introduce family medicine as an undergraduate subject reflects a holistic approach to training future doctors in effectively delivering difficult news and addressing the diverse needs of patients and their families.

### Theme 2: limited importance of breaking bad news in medical education

The medical educationists expressed that at present BBN does not hold a significant place in the teaching and training of doctors in Pakistan. The focus is primarily on technical clinical knowledge and skill development, often neglecting important soft skills such as communication skills, research skills, and logistics. This lack of emphasis on communication training implies that doctors may not be adequately prepared to handle the complexities of BBN and managing the subsequent situations effectively.

During the interviews, one medical educationist highlighted, *“In our curriculum, there is a major gap when it comes to training doctors in breaking bad news. The focus is more on technical aspects, and soft skills like communication are often overlooked. This can lead to doctors struggling in delivering difficult news and navigating the emotional complexities that follow.“*[P1].

The participants also expressed concerns about the limited exposure and opportunities for doctors to stay up to date with constantly evolving medical knowledge. They emphasized the importance of continuous professional development to ensure doctors are equipped with the latest information and best practices in BBN effectively.

One interviewee shared, *“It is crucial for doctors to have appropriate exposure to stay updated with the latest medical knowledge. Breaking bad news requires not only clinical expertise but also an understanding of the emotional and psychological aspects. Continuous professional development programs can help doctors refine their skills and keep abreast of the advancements in this field.”* [P3].

### Theme 3: learning by example and long-term impact of communication

The interviewees emphasized that BBN cannot be solely taught through theoretical instruction but should be demonstrated through practical examples and role modeling. They highlighted the significance of the communication process itself, as it can have long-term effects on the lives of patients and their families.

An interviewee emphasized, *“It’s not just about teaching the process of breaking bad news; it’s about demonstrating empathy, active listening, and providing support throughout the entire journey. Learning by example and observing experienced doctors can be invaluable in developing the necessary communication skills. We must realize that the way we communicate with people during difficult times can have a profound impact on their well-being.”* [P2].

### Theme 4: lack of standardized training and guidelines

The medical educationists highlighted the absence of standardized training programs and guidelines specifically tailored to breaking bad news in Pakistan. They emphasized the need for a structured curriculum that includes comprehensive training modules and clear guidelines to ensure consistent and effective communication when delivering difficult news.

One interviewee stated, *“There is a lack of standardized training and guidelines for breaking bad news in our medical education system. Without a structured curriculum and clear guidelines, doctors may face challenges in approaching these sensitive conversations. Establishing standardized training programs would provide doctors with the necessary tools and frameworks to navigate such situations effectively.”* [P4].

### Theme 5: inter-professional collaboration and team-based approach

The interviewees emphasized the importance of inter-professional collaboration and a team-based approach in BBN. They highlighted the need for effective communication and coordination among healthcare professionals, including doctors, nurses, psychologists, and social workers, to provide comprehensive support to patients and their families.

One medical educationist shared, *“Breaking bad news is a complex process that requires a team-based approach. It is crucial for doctors to collaborate with other healthcare professionals, such as nurses, psychologists, and social workers, to ensure holistic care and support for patients and their families. Promoting effective inter-professional communication is essential in delivering sensitive news with empathy and addressing the diverse needs of patients.”* [P7].

## Discussion

The present study aimed to explore the practices and training of clinicians in BBN to patients and their care givers in Pakistan. The combination of quantitative and qualitative findings, along with comparisons drawn from other studies conducted in developing countries, provides a comprehensive understanding of the current state of BBN practices and training in Pakistan and its relation to similar contexts.

Breaking bad news is part of the daily duties of almost all the clinicians. A study conducted in Sudan found that 56% of physicians had received training in BBN, indicating a relatively lower percentage compared to our study [15]. Similarly, a study from Ethiopia reported that 82% of participant physicians were not even aware of the SPIKES protocol, and 84% had no formal or informal training in BBN [8]. These findings suggest that the level of training and awareness regarding BBN varies across different developing countries. In our study revealed that only 9% of the participants reported receiving formal training specifically focused on BBN. This finding is consistent with studies conducted in other developing countries. For instance, a study from Lahore, Pakistan, involving postgraduate trainees, found a lack of knowledge and low satisfaction regarding BBN skills [16]. Similarly, a study in Peshawar, Pakistan, reported that 95% of participants had no training in BBN, highlighting a common gap in training among healthcare professionals [12]. Despite the fact that there is no formal training on BBN, the self-reported data in our study is quite positive.

The qualitative component of the study added valuable insights to complement the quantitative findings. Through in-depth interviews, participants’ experiences, perspectives, and challenges regarding BBN were explored. This approach provided a deeper understanding of the participants’ thoughts, emotions, and contextual factors influencing their communication practices. Themes and patterns emerged, offering a nuanced understanding of the quantitative results. The qualitative component also captured participants’ perceptions of training effectiveness, suggestions for improvement, and barriers to implementing optimal communication practices. Nonetheless, respondents were of the view that either at undergraduate or as part of the continuing education, inclusion of BBN training must be considered and that there should be a structured curriculum. However, there is an incongruent viewpoint too where some respondents said that skills of BBN come with experiential learning and maturity, and that it is about exhibiting one’s empathetic attitude and care during difficult times. This mixed methods approach allowed for a comprehensive examination of the research questions, generating practical implications for improving physician practices in breaking bad news [16, 17].

Comparisons drawn from other developing countries also highlight the need for standardized training programs and guidelines for BBN. For instance, according to one research, adherence to the SPIKES protocol varied among participants, with 35–79% claiming to follow the protocol in routine practice [15]. Similarly, a study in Ethiopia found that a significant percentage of physicians were not complying with the guidelines of BBN [17]. These findings indicate the need for structured curricula and clear guidelines to ensure consistent and effective communication skills amongst doctors [18]. The importance of paying enough attention to the emotions of the recipient and the need to provide support after breaking bad news cannot be undermined at all [19]. A cultural shift is required within the medical profession and healthcare more generally so that BBN is viewed not merely as a soft skill but a professional responsibility for the doctor and a right for the patients and families who wish to have it [20].

### Limitations

Our study has few limitations too. Very few participants were of the consultant cadre, most of the responded were junior doctors. Patients as well as the care givers are important stakeholders in this issue. Their views and perceptions were not explored in qualitative component of the study.

## Conclusion

This study offers valuable insights into the practices and training of clinicians involved in BBN in Pakistan. Comparisons with other studies conducted in developing countries reveal both similarities and differences in BBN practices and training. The findings underscore the necessity of standardized training programs, formal guidelines, and improved communication skills education within medical curricula across developing nations. Recommendations arising from this study include integrating communication skills into the medical curriculum, developing standardized training programs, promoting continuous professional development, fostering inter-professional collaboration, and recognizing the importance of communication skills. By taking these steps, healthcare professionals will be equipped with the necessary tools to navigate the complexities of breaking bad news effectively and to provide compassionate care. Collaboration among medical institutions, policymakers, and regulatory bodies is essential to prioritize communication skills training, establish clear guidelines, and emphasize the value of empathetic and effective communication. Efforts should be directed towards increasing awareness, providing comprehensive training, and emphasizing the significance of effective communication when delivering difficult news, thus ensuring optimal patient care and support during challenging situations. Implementation of these recommendations will enhance the delivery of difficult news, increase patient satisfaction, and ensure comprehensive support during challenging times.

### Electronic supplementary material

Below is the link to the electronic supplementary material.

Supplementary Material 1

## Data Availability

The datasets used and/or analyzed during the current study are available from the corresponding author on reasonable request.
