# Supplementary material for: Breaking bad news: A mix methods study reporting the need for improving communication skills among doctors in Pakistan
Source: BMC Health Serv Res. 2024 May 6;24:588. doi: 10.1186/s12913-024-11056-2 (PMC11075204; doi:10.1186/s12913-024-11056-2)
Supplement: Supplementary file 1 — Supplementary Material 1 [file 12913_2024_11056_MOESM1_ESM.pdf]

SPIKES protocol for breaking bad news

The following summary is adapted from:

- Baile, W. et al. SPIKES – A six step protocol for delivering bad news: application to the patient with cancer. The Oncologist 2000; 5:302-311.
- Buckman, R. Breaking bad news: the S-P-I-K-E-S strategy. Community Oncology 2005; 2: 183-142.

The SPIKES protocol for breaking bad news has four objectives:

- Gathering information from the patient
- Transmitting the medical information
- Providing support to the patient
- Eliciting patient's collaboration in developing a strategy or treatment for the future.

Strategy for breaking bad news

Meeting the above goals can be accomplished by completing six tasks, each of which is associated with specific skills.

Six Steps of SPIKES:

S – Setting

- Arrange for some privacy
- Involve significant others
- Sit down
- Make connection and establish rapport with the patient
- Manage time constraints and interruptions.

P – Perception of condition/seriousness

- Determine what the patient knows about the medical condition or what he suspects.
- Listen to the patient's level of comprehension
- Accept denial but do not confront at this stage.

I – Invitation from the patient to give information

- Ask patient if s/he wishes to know the details of the medical condition and/or treatment
- Accept patient's right not to know
- Offer to answer questions later if s/he wishes.

K – Knowledge: giving medical facts

- Use language intelligible to patient
- Consider educational level, socio-cultural background, current emotional state
- Give information in small chunks
- Check whether the patient understood what you said

- Respond to the patient's reactions as they occur
- Give any positive aspects first
  e.g.: Cancer has not spread to lymph nodes, highly responsive to therapy, treatment available locally etc.
- Give facts accurately about treatment options, prognosis, costs etc.

E - Explore emotions and sympathize

- Prepare to give an empathetic response:

1. Identify emotion expressed by the patient (sadness, silence, shock etc.)
2. Identify cause/source of emotion
3. Give the patient time express his or her feelings, then respond in a way that demonstrates you have recognized connection between 1 and 2.

S – Strategy and summary

- Close the interview
- Ask whether they want to clarify something else
- Offer agenda for the next meeting

  eg: I will speak to you again when we have the opinion of cancer specialist.
